# Angina Pectoris without Lesion of the Coronary Arteries

**Published:** 1887-08

**Authors:** 


### ANGINA PECTORIS WITHOUT LESION OF THE CORONARY ARTERIES.

M. BALL reports to the Société des Hôpitaux a case of thiskind. The patient was a man 34 years of age, who had previously had three attacks of rheumatism and one of intermittent fever. The attacks of angina began five weeks before death. There was slight aortic insufficiency, persistent and severe headache. The patient was a smoker. The autopsy showed the orifices of the coronary arteries permeable, and even dilated; insufficiency of the aortic opening; dilatation of the aorta, and hypertrophy of the left ventricle, whilst the right appeared diminished, and, as it were, crushed by the left. M. Ball explained the death by the imperfect action of the right ventricle. The quantity of blood emptied into the right side of the heart by the pulmonary veins being lessened, there was cardiac ischemia and a deficient circulation, not because of any narrowing of the coronary arteries, but despite of their dilatation. Commenting on the case, M. Huchard said he thought it one of tobacco angina, in which the patient died of arterial spasms as described by Cl. Bernard.—*Le Concours Médical, June 4, 1887.*

